# Vaccination in the Light of the Royal British Commission

**Published:** 1899-10

**Authors:** M. R. Leverson

**Affiliations:** Fort Hamilton, L. I., N. Y.


### VACCINATION IN THE LIGHT OF THE ROYAL BRITISH COMMISSION.

M. R. LEVERSON, M. D., FORT HAMILTON, L. I., N. Y.

(Continued from May No., p. 228.)

Dr. Charles Creighton began his testimony on the 4th of December, 1889. It was continued on the 11th of December, on the 22d of January, and on the 29th of January, 1890.

Qs. 4,181-7. He is a doctor of medicine; has been occupied since 1872 with original laboratory work on pathology and the allied sciences; also with teaching anatomy. He has translated a treatise of Professor Hirsch on "Geographical and Historical Pathology;" has written papers on pathological subjects, and since 1886 has made a special study of vaccination and small-pox.*

Q. 4,818. He began by inquiring into the nature of small-pox and the historical sources of vaccine lymph, and (Q.

* He is the author of the article on "Vaccination" in the new (ninth) edition of the *Encyclopedia Brittanica*, of "Cow-pox and Vaccinal Syphilis," and of " Jenner and Vaccination."

4,819) has also investigated the subject from its statistical aspect, but has not written upon that part of the question.

Then (Q. 4,820) with regard to the historical sources of British vaccine; the first put into circulation was that of Woodville, which became the source of a continuous series of vaccinations and was of a much milder type than that tried experimentally by Jenner, from which he did not succeed in raising a stock (Q. 4,824).

Qs. 4,821-2. There were two sources employed by Woodville, one being directly from diseased teats of cows, and the other from disease contracted by milkers through handling diseased teats.

Q. 4,825. The point this illustrates is the original characteristics of cow-pox as Jenner experienced them, and as they have been found by other observers.

Q. 4,826. Woodville's success was owing to the fact that the lymph started was taken from a milder type of cow-pox than that of Jenner.

Qs. 4,828-9. The characteristics of cow-pox, as it occurs in the cow, as loosely described by Jenner and more particularly by one of his veterinary neighbors and contemporaries who practiced in Gloucester,* were that the disease was an ulcerous disease on the teats, and for the most part confined to them; it occurred only in a cow in milk, and it was supposed to begin in one particular animal and to be carried by the hand of the milker to other animals. No medical man seems ever to have seen the disease in its earlier stages. No person capable of describing the initial steps of the process ever saw those steps, so far as it known. The milkers were the sole authorities as to the first appearances of the disease. Whenever the affected teats of a cow were seen by medical men

---

*Mr. Clayton, whose description of it was published by Dr. Beddoes in his "Contributions to Physical and Medical Knowledge," Bristol, 1799, p. 387.—M. R. L.

or veterinarians, they were in a state of ulceration covered by crusts. The disease, wherever it has been seen by any one capable of describing it, has been described as an ulcerous affection of the paps for the most part covered by large crusts; sometimes the crusts have been less adherent than at other times, in which case one sees the ulcerous nature of the process quite distinctly; large ulcers described by Ceely forty years after as having hard, round, sloping edges, and sometimes with granulations rising from the floor of the ulcer. Ceely says he never could get lymph from unbroken vesicles. Mr. Clayton, who described the disease with more precision than Jenner, said: "This disease may arise from any cause irritating or excoriating the teats, but the teats are often chapped without the cow-pox succeeding. In chaps of the teats they generally swell, but in the cow-pox the teats seldom swell at all, but are gradually destroyed by ulceration."

Q. 4,830. Ceely adopted this view that the cow-pox arises on the top of casual cracks or other common ailments of the cow paps, and has practically accepted the view that it arose spontaneously. "Spontaneously" is not an accurate term, but it was that generally used. It was used by Jenner in his evidence before the Parliamentary Committee of 1802.

Q. 4,831. By "spontaneous" was meant that it was not derived by contact with any other animal or with a human being.

Q. 4,832. The milker's cow-pox was thus described by Jenner. After describing their beginning, he says:

"These symptoms, varying in their degrees of violence, generally continue from one to three or four days, leaving ulcerated sores about the hands, which, from the sensibility of the parts, are very troublesome and commonly heal slowly, frequently becoming phagedenic, like those from whence they sprung. The lips, nostrils, eyelids, and other parts of the body are sometimes affected with sores, but these evidently arise from their being heedlessly rubbed or scratched with the patient's infected finger." He then gave references to some sixteen cases which had been infected, and now and then mentioned the nature of their sores; for instance, he speaks of corroding ulcers* in one case; in another, of "a poor girl who produced an ulceration on her lip by frequently holding her finger to her mouth to cool the raging of a cow-pox sore by blowing upon it."†

Q. 4,833. Ceely confirms this account, which is published in the eighth and tenth volumes of "The Transactions of the Provincial Medical and Surgical Association." He gave colored plates of the sores upon the milker's hands, and one on the temple in which the sore was three-quarters of an inch long.

Q. 4,834. Ulceration was a common sequel of the original vaccinations, and Dr. Creighton believes it was the real barrier to his establishing a stock for general use. The instances of ulceration following vaccination in the first removes from the cow are so numerous and uniformly recorded by observers in all countries that Dr. Creighton thinks it unnecessary to mention particular cases, but quotes from a pamphlet by Mr. Hicks, an intimate friend of Jenner, and a strong supporter of his practice.

Q. 4,835-6. Quoting from Mr. Hicks's pamphlet, published at Stroud, 1803, p. 43: "Large ulcers are often the consequences of such casual affection (*i. e.*, accidentally in the case of milkers), and the virus may probably be rendered more mild after having passed several times through the human constitution. This, I think, is the only satisfactory way in which we can account for the fact of sore arms being so prevalent in the first stages of vaccine inoculation, and for their having almost entirely disappeared since." The same

* Case XI, William Stinchcomb, "An Inquiry, etc.," Jenner.

† Ubi., Sup., p. 50. Idem, Vol. II, of Crookshank's "History and Pathology of Vaccination," p. 25.

frequency of ulcerations in the early practice was noticed at Rotterdam, at Brandenburg, at Milan, and at other places abroad which I cannot now enumerate, as well as frequently in England. But in Woodville's cases there seems to have been only one in which ulceration followed.

Q. 4,837. At the end of 1798 Jenner had no lymph to supply those who wrote to him for it. In January, 1799, Woodville heard of cow-pox at a cow-house in Gray's Inn Lane, and by a remarkable piece of luck managed to start a stock of lymph which went on continuously for thirty or forty years, and may be going on still.

Q. 4,838 (Sir James Paget). "You admit the element of luck?" "A large element of it. Perhaps I might exp'ain wherein I think the luck consisted. Woodville got notice of the disease very soon after it had broken out in Gray's Inn Lane, so that he obtained matter from those cows at an early stage of the disease, and when only two or three of the cows had become infected and when it was comparatively new and had not become cultivated into a more inveterate and virulent type."

Q. 4,840 (Professor Michael Foster). "It must have been an ulcer, I think; it must have been an ulcer under a crust."

Q. 4,841 (Chairman). "Why do you think so?" "Because no one sees it in the cow in any other form."

